# Protein- and nutrient-normalized environmental footprints of university canteen meals

**DOI:** 10.3389/fnut.2026.1810180

**Published:** 2026-05-14

**Authors:** Merve Çapaş, Sukru Taner Azgin

**Affiliations:** 1 Department of Nutrition and Dietetics, Faculty of Health Sciences, Erciyes University, Kayseri, Türkiye; 2 Sustainability Coordination Office, Erciyes University, Kayseri, Türkiye; 3 Department of Environmental Engineering, Faculty of Engineering, Erciyes University, Kayseri, Türkiye

**Keywords:** carbon footprint, environmental footprint, institutional food services, life cycle assessment, nutrient-based normalization, protein-based normalization, sustainable diets, water footprint

## Abstract

**Background:**

The environmental sustainability of institutional diets is increasingly recognized as a core concern in nutrition science, as food service settings influence population-level dietary patterns. University canteens offer a strategic opportunity to align nutritional quality with environmental sustainability through evidence-based menu planning.

**Objectives:**

This study assessed the carbon and water footprints of meals served in a large university canteen system using a life cycle assessment (LCA) approach, integrated with nutritional composition analysis and secondary protein-based normalization to inform sustainable diet planning.

**Methods:**

A total of 150 standardized recipes served in Erciyes University canteens in 2023 were evaluated. Ninety-five individual ingredients were modeled using SimaPro within a cradle-to-kitchen system boundary. Nutritional composition was calculated using the BEBİS database. Environmental impacts were first calculated per standardized serving using a cradle-to-kitchen LCA framework, then compared across meal groups and seasons; additionally, results were secondarily normalized by energy and protein content to explore nutrient-informed environmental efficiency.

**Results:**

Significant differences were observed among meal groups for both carbon and water footprints (*p <* 0.001). Meat-based dishes, pastries, desserts, and yogurt-based meals exhibited the highest average carbon footprints (up to approximately 1.4 kg CO₂e per portion), whereas legume-based dishes, salads, olive oil–based vegetable meals, and compotes consistently showed the lowest values (<0.4 kg CO₂e). Seasonal variation in carbon footprint was statistically significant but modest (*p* = 0.032), with higher values observed in autumn and lower values in summer, while nutritional composition remained relatively stable across seasons. Energy-based normalization reduced between-group differences, whereas protein-based normalization amplified contrasts, identifying legumes as the most environmentally efficient protein sources.

**Conclusion:**

Meal composition—particularly protein source and nutritional density—is a primary determinant of environmental impact in university food services. Incorporating protein- and nutrient-based normalization into LCA-based environmental assessments represents a conceptual advance by linking environmental performance directly to nutritional function, thereby supporting nutritionally informed strategies for sustainable menu planning in institutional settings.

## 1 Introduction

Food production and consumption are widely recognized as significant contributors to global environmental challenges. Among these, greenhouse gas emissions are particularly substantial, with food systems accounting for around one-third of total global emissions (1, 2). Climate change, environmental degradation, and threats to public health are directly linked to the sustainability of food systems (3). In this context, transitioning to sustainable dietary systems offers environmental benefits and the potential to enhance both individual and public health (4).

Large-scale food services in institutional settings such as hospitals, schools, and universities generate significant environmental impacts. In these settings, where thousands of meals are regularly prepared, the use of raw materials, energy consumption, and water usage directly contributes to environmental burdens, while the resulting waste further amplifies these impacts (5). Universities, in particular, are well placed to intervene due to their ability to manage procurement and meal production processes directly, enabling them to encourage behavioral changes among students in the short and long term and to educate them on this matter (6–9). In line with the Sustainable Development Goals (particularly SDGs 12 and 13, which focus on responsible consumption and production and climate action, respectively), it is of critical importance to take strategic steps to identify, assess, and mitigate environmental impacts within mass catering systems (MCS) (10).

Conducting water and carbon footprint analyses throughout all stages of meal pro-duction and consumption in MCS represents a tangible and crucial step for identifying environmental impacts and developing strategies to mitigate them (11, 12). In this context, the carbon footprint functions as a vital indicator, facilitating the quantitative assessment of the environmental impacts generated throughout the production process of a food item. This enables a more concrete, detailed evaluation of a product or service’s adverse effects on the ecosystem and facilitates comparative analyses. The quantification of the impact of human activities on the environment is achieved by calculating the carbon dioxide equivalent (CO₂e) of all processes, including production, transportation, and consumption, that directly or indirectly contribute to greenhouse gas emissions (12).

Food systems represent a primary sector that poses a significant threat to environmental sustainability. This is primarily due to the substantial greenhouse gas emissions generated during key stages such as agriculture, food processing, cooking, and waste management (11, 13). Similarly, the water footprint constitutes a pivotal indicator, reflecting the aggregate volume of freshwater consumed directly or indirectly as a consequence of human activities (14, 15). The water footprint of an individual, community, or organization is defined as the total volume of freshwater used in the production processes of the goods and services consumed by that particular entity (16, 17). As indicated by the extant literature, these indicators play a crucial role in assessing the environmental performance of food systems and in designing effective sustainability strategies (12, 14). It is therefore considered that they are among the fundamental tools for evaluating MCS.

In view of the unsustainable nature of contemporary global dietary trends and the mounting concerns regarding food security, there is an urgent need to assess and improve the environmental profiles of menus offered in MCS. In this context, prioritizing ecologically responsible, fair, accessible, locally sourced, healthy, safe, and low-waste foods in daily diets is of significant importance. This approach is further supported by balanced menu planning, characterized by its high carbohydrate content, abundant dietary fiber, substantial plant-based protein, and adequate micronutrient intake (3, 18, 19). Furthermore, it is imperative to educate consumers about the importance of responsible, conscious food consumption and the prevention of food waste caused by excessive purchasing and over-preparation. This is of paramount importance in achieving sustainable development goals.

In this regard, significant contributions can be made toward targets such as SDGs 2: Zero Hunger, SDGs 4: Quality Education, SDGs 12: Responsible Consumption and Production, and SDGs 13: Climate Action (20, 21). Moreover, as consumer awareness of the environmental impacts of food choices increases, restaurants and foodservice providers have begun restructuring their offerings to meet growing demands for sustainability (21). In this context, the Life Cycle Assessment (LCA) method has become a widely used tool in the food sector for identifying environmentally and energetically critical areas and ensuring alignment with sustainable development goals (22–24). LCA is a methodology defined by the International Organization for Standardization (ISO) 14,040 and 14,044 standards that systematically analyzes the entire life cycle of a product or service—from raw material extraction through production, distribution, consumption, and final waste management—following a “cradle-to-grave” approach (25). In the context of MCS, this approach facilitates a comprehensive evaluation of environmental impacts of processes such as raw material production, energy and water consumption, cooking methods, and waste management, thereby supporting decision-making processes for sustainable menu planning (26). Therefore, in this study, the environmental performance of MCS was comprehensively analyzed using LCA methodology.

The complex and multi-layered nature of MCS is characterized by supply chains that encompass all intermediate stages, including food production, preparation, cooking, distribution, and waste management. On average, producing a single meal is estimated to generate 1.43–1.67 kg of CO₂e emissions (27, 28), with a significant proportion of these emissions attributed to meat and dairy products. These products have been re-ported to contribute substantially to total greenhouse gas emissions, accounting for approximately 50% of the climate change impact associated with MCS (29). The production of animal-based products is considered a primary source of methane, accounting for approximately 37% of total greenhouse gas emissions (30–32) and representing a highly water-intensive activity, contributing to around 30% of global freshwater consumption (33).

In recent years, there has been a noticeable increase in the number of studies focusing on MCS in international literature. Specifically, research undertaken in the context of school, university, and workplace canteens has focused on calculating the carbon and water footprints of menus and on proposing more sustainable menu-planning strategies based on these findings (4, 5, 34–47). Despite a considerable body of research evaluating the environmental impacts of food products (48–50), the preponderance of this research has focused on a single product or a restricted range of food items (51–56). Conversely, although several systematic reviews have analyzed greenhouse gas emissions from a wide range of fresh foods (57, 58), the majority of these studies have over-looked the nutritional dimension. Consequently, the extant literature has either remained limited in terms of product diversity in environmental impact assessments or neglected the nutritional dimension altogether. This underscores the need for a more comprehensive, multidimensional evaluation approach.

Nevertheless, a significant finding consistently emphasized across studies is that the environmental footprint of animal-based protein sources is higher than that of plant-based foods. In contrast, the evidence suggests that plant-based diets can lead to a significant reduction in greenhouse gas emissions and water footprints (3, 12, 18, 19, 34, 59). A study conducted in Türkiye found that the current menus offered in public university hospitals have a high carbon and water footprint. Furthermore, it was found that revising these menus in line with the principles of the Turkish Dietary Guidelines and the Mediterranean Diet could reduce their environmental impacts by 30–50% (4).

In this context, the present study provides a comprehensive analysis of the carbon and water footprints of menus served at Erciyes University canteens throughout 2023 using the LCA approach. By accounting for a range of factors, including ingredient se-lection, cooking methods, and energy and water consumption, the research offers a more comprehensive and holistic perspective than similar studies in the literature. In this respect, the study quantifies the environmental impacts and provides a scientific framework for sustainable menu planning and the development of sustainability strategies.

## 2 Materials and methods

This study was conducted between January and December 2023 with the primary objective of quantifying the carbon and water footprints of meals served in the cafeterias of Erciyes University using LCA methodology. The primary aim of the research endeavor is to conduct a rigorous evaluation of the environmental implications associated with institutional meal services, with a particular focus on their carbon and water footprints. This objective is driven by the overarching ambition to establish a robust scientific foundation for the development and implementation of more sustainable menu planning strategies (60–62).

The present study analyzes lunch menus provided to students and staff throughout 2023. The analysis encompasses 150 recipes and 95 distinct food ingredients used in these meals. The menus were devised by institutional dietitians guided by the principles of healthy, balanced nutrition, with consideration for dietary diversity and overall nutritional quality. The food items on the menus were systematically classified into five major categories: starch-based products, fruits and vegetables, milk and dairy products, non-dairy protein sources (meat, poultry, fish, legumes, eggs), and fats and oilseeds (Table 1).

**Table 1:** Classification of food items used in the university canteen menus according to food groups.

Starch-based products	Fruits and vegetables			Milk and dairy products	Non-dairy protein sources	Fats and oilseeds
Wheat flour	Broccoli	Sour cherry	Cucumber	Cow Milk	Beef (Cubed)	Sunflower oil
Bulgur	Iceberg lettuce	Green pepper	Strawberry	Yogurt	Chicken thigh	Tail fat
Wheat for ashure	Dried fig	Zucchini	Corn	Kashar cheese	Diced chicken	Frying oil
Rice	Arugula	Banana	Red Cabbage	White cheese	Chicken chop	Butter
Cut pasta	Dried apricot	Garlic	Apple	Tulum cheese	Whiting fillet (Frozen)	Olive oil (Riviera)
Vermicelli	Parsley	Dill	Curly Lettuce	Strained Yogurt	Egg	Baklava oil
Noodle	Fresh beans	Mandarin	White Cabbage		Chickpeas	Pastry margarine
Tarhana (Powder)	Dried grapes	Carrot	Plum		Green lentils	Walnut
Pasta	Green peas	Red Pepper	Purslane		Yellow lentils	Rice hazelnut
Fine bulgur	Pomegranate	Orange	Stuffing pepper		Red lentils	Pistachio
Starch	Onion	Mint	Watermelon		Dried beans	Ground pistachio
Granulated sugar	Lemon	Spinach	Long Green pepper		Kidney beans	
Semolina	Potato	Peach	Melon			
Poppy seed	Quince	Mushroom	Cherry			
Rice flour	Tomato	Cauliflower				
Shredded pastry (Kadayif)	Eggplant	Pear				

The data on menu composition were collected in close collaboration with cafeteria staff, and detailed records were maintained for each food item, including its quantity, origin, supplier information, distribution method, and cooking technique (cooking time, equipment used, and energy source). The LCA process was conducted in accordance with ISO 14040 and ISO 14044 standards. It consisted of four main stages: The following four stages were identified as being integral to the research process: (i) the establishment of objectives and the definition of the scope, (ii) the analysis of the life cycle inventory (LCI), (iii) the assessment of the impact, and (iv) the interpretation of the results. The system boundary was defined using a “cradle-to-kitchen” approach, encompassing production, distribution, and cooking stages within the analysis. This boundary was selected to capture the main upstream and operational processes directly associated with institutional meal provision under standardized service conditions and to ensure comparability across meal groups. The primary functional unit used in the LCA modeling was one standardized serving (portion) of each menu item as prepared and served in the university canteens. Accordingly, all environmental impacts were first calculated on a per-portion basis within the defined system boundary. Nutritional variables, including energy (kcal) and macronutrient content (g), were not used as the primary functional unit; rather, they were applied as secondary normalization metrics to evaluate environmental impacts under nutritionally comparable conditions. In particular, energy- and protein-based normalization were used as complementary analytical approaches to examine nutrient-informed environmental efficiency across meal groups.

During the inventory analysis, a comprehensive compilation was conducted of all raw materials, energy consumption, waste generation, and emissions associated with the meal production process. These data were then modeled using SimaPro 9.0 software. The background data were derived from the Ecoinvent® 3.5 database. Water footprint assessment was conducted within a single accounting framework, focusing on the blue and green water components of food items. Ingredient-level water footprint values were assigned based primarily on published datasets and methodological references, particularly those of Hoekstra and Mekonnen (33), with additional support from the Water Footprint Network (63) where necessary. References such as González-García et al. (64) were used to support the application of water footprint accounting in meal-based contexts rather than to define a separate calculation framework. Blue and green water components were evaluated separately for each ingredient, taking into account its geographical origin where relevant, and were then aggregated at the recipe level according to standardized portion composition. The water footprint indicator was therefore calculated solely from the blue and green components, consistent with the scope of the present study and previous applications in the literature (65, 66). Although minor differences may exist among published sources in terms of geographical coverage and background assumptions, the same water footprint accounting logic was applied consistently across all ingredients and recipes to preserve internal comparability.

The Intergovernmental Panel on Climate Change (IPCC) 2013 methodology was employed to calculate the carbon footprint, with total greenhouse gas emissions generated across the product life cycle being expressed in kilograms of CO₂e (kg CO₂e). A comprehensive dataset from five distinct LCA studies, focusing on the production phase, was used to analyze 95 food ingredients incorporated into the menu. The analysis was conducted utilizing a cradle-to-kitchen approach. Given the unavailability of Türkiye-specific datasets, it was hypothesized that food ingredients were sourced from various countries. Consequently, the CML-IA baseline V3.10/world 2000 method was applied. In the cooking phase, a range of standard culinary practices were considered, including boiling, frying, stewing, and baking. The energy inputs for these activities were calculated in kilowatt-hours (kWh) using the “natural gas burned in gas turbine (TR)” process serving as the reference point. For electrically powered cooking equipment, electricity use was converted into equivalent cooking energy demand to harmonize energy accounting across different kitchen technologies within the modeling framework. This standardized representation was used to maintain consistency in the treatment of the cooking stage across recipes. However, because electricity and natural gas are environmentally distinct energy carriers, this simplification may introduce some uncertainty into the absolute footprint estimates of the cooking phase; accordingly, the cooking-stage results should be interpreted primarily as comparative approximations rather than technology-specific emission factors (67, 68). Spices and flavoring agents, whose quantities were negligible and had minimal impact on total environmental outcomes, were excluded from the analysis. In cases where Türkiye-specific ingredients were not available in the LCA database, analogous alternatives were used (green lentils instead of peas, tomato sauce instead of tomato paste, wheat instead of bulgur, and almonds instead of walnuts). These substitutions may have introduced some uncertainty in the absolute footprint estimates; however, because they were limited in number and applied consistently within the modeling framework, they are unlikely to have materially affected the relative comparisons or overall ranking patterns across recipes and meal groups (25, 48).

**Figure 1:**
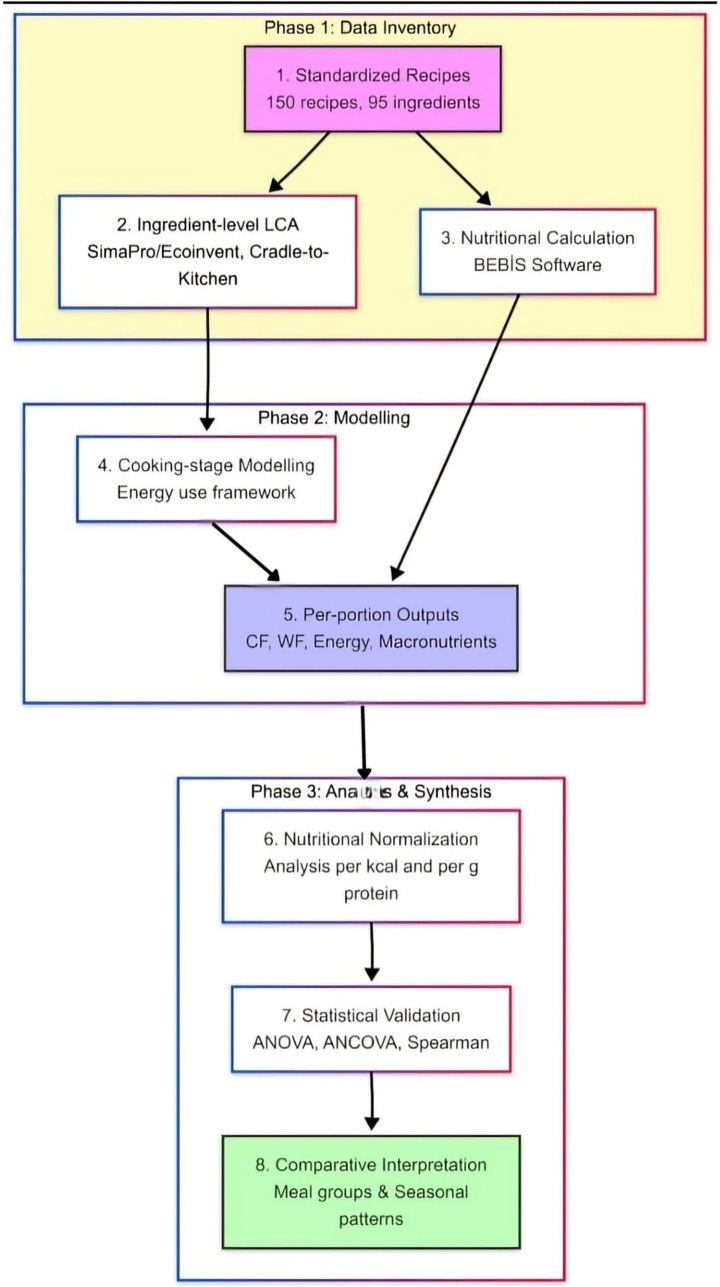
Analytical workflow of the study. The figure summarizes the main analytical stages, including standardized recipe compilation, ingredient-level LCA modeling, cooking-stage energy modeling, BEBİS-based nutrient calculation, normalization by energy and protein content, and statistical analysis.

Upon completion of the LCA, the resulting carbon and water footprint data were transferred to Microsoft Excel for further calculations at daily, monthly, and seasonal levels. For each dish, the relevant LCA-derived factors were incorporated to perform detailed footprint calculations, and monthly averages were used to facilitate seasonal comparisons. Nutrient composition was calculated using the Nutrition Information System (BEBİS) software based on the standardized cafeteria recipes of Erciyes University. For each menu item, ingredient quantities and units were entered according to the standard recipe formulation, and nutrient values were calculated at the recipe level and then expressed per standardized serving. In addition, one standard white bread roll routinely served with each meal was included in the nutritional calculations, in accordance with the Turkish Nutrition Guide (69). Accordingly, the reported energy and macronutrient values reflect portion-based nutrient content rather than unstandardized ingredient listings. All nutritional values were therefore derived from standardized recipe quantities rather than from the mere presence or absence of ingredients in a recipe. The adequacy of the menus was assessed by evaluating the proportion of daily energy and nutrient requirements met by lunch. The energy requirements of the subject were calculated based on a sedentary lifestyle, using data from the Türkiye Nutrition and Health Survey (70). The analytical workflow consisted of five sequential stages: (i) compilation of standardized recipes and ingredient inventories; (ii) assignment of ingredient-level environmental data and modeling of cooking-stage energy use; (iii) calculation of nutrient composition per standardized serving using BEBİS; (iv) normalization of environmental indicators by energy and protein content; and (v) statistical evaluation using descriptive analysis, correlation analysis, ANOVA, and ANCOVA. The overall analytical workflow of the study is summarized in Figure 1.

Statistical analyses were performed using IBM SPSS Statistics software (Version 22.0; IBM Corp., Armonk, NY, USA). Nutritional variables (energy, carbohydrate, protein, and fat) and environmental indicators (carbon and water footprints) were summarized using descriptive statistics and are presented as means ± standard deviations. Data normality was assessed using the Kolmogorov–Smirnov test, and homogeneity of variances was evaluated with Levene’s test. Based on these assumptions, group comparisons were conducted using one-way analysis of variance (ANOVA) with Games–Howell *post hoc* tests, as appropriate. Associations between nutritional variables and environmental indicators were examined using Spearman’s rank correlation analysis. ANCOVA was used to compare carbon and water footprint values across meal groups while statistically controlling for energy and protein content as covariates. In these models, meal group was treated as the fixed factor, environmental footprint indicators were treated as dependent variables, and energy and protein values were entered as covariates to evaluate adjusted between-group differences under nutritionally comparable conditions. The resulting adjusted estimates were interpreted as model-based comparative values rather than directly observed physical measurements. Statistical significance was set at *p <* 0.05.

## 3 Results

### 3.1 Nutritional and environmental characteristics of meals served in the university canteens

**Figure 2:**
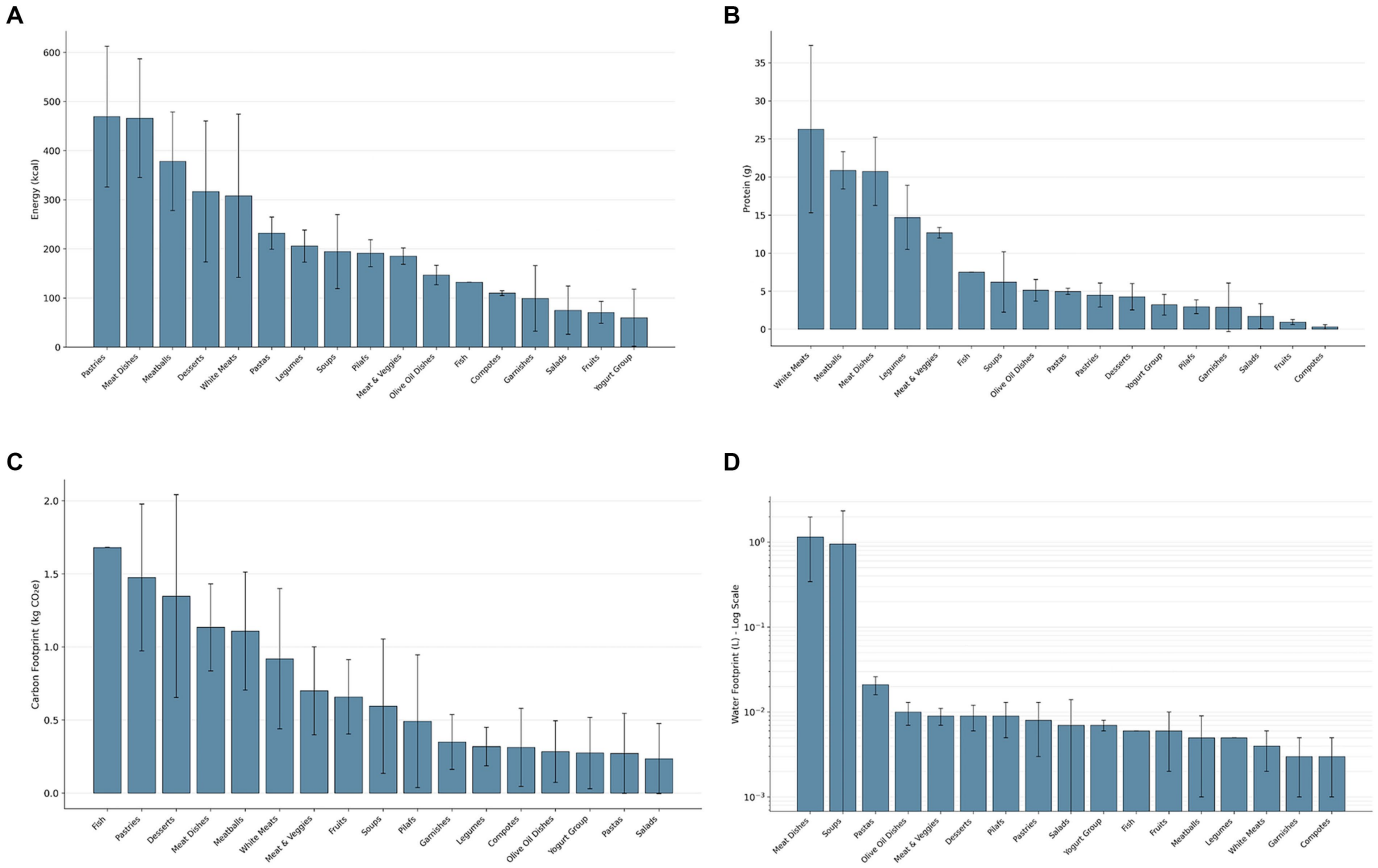
Comparative nutritional and environmental profiles of meal groups. Panels display mean ± standard deviation values of **(A)** energy (kcal), **(B)** protein (g), **(C)** carbon footprint (kg CO*_2_*-eq), and **(D)** water footprint (L). In each panel, meal groups are independently ranked in descending order based on their respective mean values to highlight the highest and lowest contributors within each metric. Note that water footprint values in Panel (D) are presented on a logarithmic scale to facilitate visual comparison across diverse magnitudes.

The environmental and nutritional profiles of meals served at Erciyes University canteens in 2023 exhibited significant variation across meal groups (Table 2). A comprehensive analysis encompassing energy, carbohydrate, protein, fat, carbon footprint, and water footprint indicators revealed significant disparities between the groups (*p <* 0.001). These findings underscore the heterogeneity in composition and sourcing, highlighting the necessity for a multifaceted approach to environmental impact assessment. These differences are visually summarized across four panels in Figure 2, representing nutritional and environmental indicators (Figures 2A–D).

**Table 2:** Nutritional composition and environmental footprints of meal groups.

Meal group	Energy (kcal)	Carbohydrate (g)	Protein (g)	Fat (g)	Carbon footprint (kg CO₂e)	Water footprint (L)
Soups	194.54 ± 75.31	18.88 ± 9.95	6.21 ± 3.96	9.43 ± 3.21	0.59 ± 0.46	0.955 ± 1.366
Meatballs	378.23 ± 100.28	24.52 ± 11.76	20.87 ± 2.46	21.92 ± 4.87	1.11 ± 0.40	0.005 ± 0.004
Meat and veggies	185.10 ± 16.58	5.33 ± 1.56	12.68 ± 0.70	11.87 ± 2.89	0.70 ± 0.30	0.009 ± 0.002
Pastas	231.92 ± 33.06	15.36 ± 1.23	4.99 ± 0.41	16.86 ± 3.82	0.27 ± 0.27	0.021 ± 0.005
Salads	75.11 ± 49.19	4.48 ± 6.27	1.71 ± 1.63	4.91 ± 3.06	0.24 ± 0.24	0.007 ± 0.007
Meat dishes	465.79 ± 120.75	24.84 ± 18.46	20.74 ± 4.48	32.01 ± 12.28	1.13 ± 0.30	1.151 ± 0.810
White meats	307.92 ± 166.18	8.85 ± 7.76	26.29 ± 10.99	19.31 ± 12.99	0.92 ± 0.48	0.004 ± 0.002
Legumes	205.69 ± 32.69	19.74 ± 4.97	14.69 ± 4.21	10.15 ± 0.03	0.32 ± 0.13	0.005 ± 0.000
Garnishes	99.14 ± 66.69	10.70 ± 6.41	2.88 ± 3.19	4.57 ± 3.81	0.35 ± 0.19	0.003 ± 0.002
Pastries	469.17 ± 143.23	25.48 ± 11.94	4.49 ± 1.57	29.11 ± 10.02	1.48 ± 0.50	0.008 ± 0.005
Fishes	132.00 ± 0.00	1.60 ± 0.00	7.50 ± 0.00	9.70 ± 0.00	1.68 ± 0.00	0.006 ± 0.000
Yogurt	59.73 ± 58.04	6.22 ± 1.00	3.22 ± 1.36	1.76 ± 0.18	0.27 ± 0.24	0.007 ± 0.001
Desserts	316.75 ± 143.57	49.05 ± 26.40	4.25 ± 1.74	10.61 ± 7.68	1.35 ± 0.69	0.009 ± 0.003
Pilafs	191.02 ± 27.32	7.82 ± 3.61	2.96 ± 0.91	7.07 ± 0.09	0.49 ± 0.45	0.009 ± 0.004
Olive oil dishes	146.71 ± 19.70	7.66 ± 3.09	5.12 ± 1.43	14.21 ± 2.03	0.28 ± 0.21	0.010 ± 0.003
Compotes	109.75 ± 4.78	30.43 ± 4.40	0.31 ± 0.30	0.28 ± 0.07	0.31 ± 0.27	0.003 ± 0.002
Fruits	70.56 ± 22.23	17.52 ± 5.36	0.95 ± 0.33	0.28 ± 0.10	0.66 ± 0.25	0.006 ± 0.004
*p*-value	*p <* 0.001	*p <* 0.001	*p <* 0.001	*p <* 0.001	*p <* 0.001	*p <* 0.001

The mean energy content of meals was highest in pastries (~469 kcal), meat-based main dishes (~466 kcal), and meatballs (~378 kcal), with desserts (~317 kcal) ranking fourth. Conversely, yoghurt-based products (~60 kcal), fruits (~71 kcal), and salads (~75 kcal) exhibited the lowest energy values. The carbohydrate content varied significantly across meal types. Desserts and compotes had the highest carbohydrate levels, averaging approximately 49 g and 30 g, respectively. In contrast, fish dishes, salads, and meat-and-vegetable dishes exhibited the lowest carbohydrate levels, averaging approximately 1.6 g, 4.5 g, and 5.3 g, respectively. The protein content was highest in white meat dishes (~26 g), meatballs, and other meat-based items, whereas compotes, fruits, and salads provided minimal amounts. A comparable trend was observed for fat content, with meat-based meals (~32 g) and pastries (~29 g) exhibiting the highest concentrations. The fat content of compotes and fruits was negligible, while that of yoghurt-based products and salads was comparatively low.

Furthermore, environmental factors exhibited significant variation between meal categories. The highest carbon footprints were observed in fish dishes (~1.68 kg CO₂e), pastries (~1.48 kg CO₂e), desserts (~1.35 kg CO₂e), and meat-based meals (~1.13 kg CO₂e). Lower values were recorded for salads (~0.24 kg CO₂e), yoghurt-based products, olive oil dishes, pilafs, and compotes. The elevated footprint in the fish category may be attributed to factors such as long-distance supply chains, cold storage, and high emission intensity. Water footprint analysis showed that meat-based meals had the highest average value at 1.15 L, followed by soups at 0.96 units. In contrast, side dishes, compotes, and white meat dishes demonstrated lower water utilization. Due to the wide range of values, water footprint results are presented on a logarithmic scale (Figure 2D).

It was evident that meals comprising higher proportions of animal-based ingredients and refined carbohydrates tended to demonstrate higher energy, carbon, and water metrics. Conversely, those based on plant ingredients and minimally processed items were associated with lower levels across all indicators (Figures 2A–D).

### 3.2 Seasonal patterns in nutritional content, carbon footprint, and water use of meals

Seasonal analyses indicated that the nutritional composition of meals remained largely stable throughout the year (Table 3).

**Table 3:** Seasonal variations in nutritional composition and ecological footprint of foods.

Parameter	Spring (*n =* 267)	Summer (*n =* 261)	Autumn (*n =* 273)	Winter (*n =* 276)	*p*-value
Energy (Kkal)	214.0 ± 146.2	216.0 ± 148.2	211.9 ± 150.8	207.7 ± 139.7	0.923
Carbohydrate (g)	19.50 ± 17.22	19.66 ± 16.32	19.47 ± 17.05	20.13 ± 18.04	0.969
Protein (g)	7.95 ± 8.21	7.97 ± 8.35	8.42 ± 9.39	7.91 ± 8.45	0.890
Fat (g)	11.47 ± 9.89	11.78 ± 10.36	11.57 ± 10.27	10.71 ± 8.91	0.608
Carbon footprint (kg CO₂e)	0.719 ± 0.551	0.704 ± 0.552	0.820 ± 0.529	0.796 ± 0.537	0.032
Water footprint (L)	0.268 ± 0.804	0.236 ± 0.715	0.197 ± 0.642	0.208 ± 0.667	0.654

The energy content of the food did not differ significantly across seasons (*p* = 0.923), with comparable mean values observed in spring (214.0 kcal), summer (216.0 kcal), autumn (211.9 kcal), and winter (207.7 kcal). Pairwise comparisons confirmed that energy values remained consistent across all seasons. Similarly, the carbohydrate content exhibited no substantial seasonal fluctuations (*p* = 0.969). The mean carbohydrate values were closely aligned across spring (19.50 g), summer (19.66 g), autumn (19.47 g), and winter (20.13 g), with no statistically significant differences observed between the seasons. The protein content exhibited stability across seasons, as indicated by a *p*-value of 0.890. The mean protein values were comparable in spring (7.95 ± 8.21 g), summer (7.96 ± 8.35 g), autumn (8.42 ± 9.39 g), and winter (7.91 ± 8.45 g), indicating no seasonal differences in protein provision. In accordance with these findings, the fat content exhibited no significant variation across seasons (*p* = 0.608). The mean fat values remained consistent across all seasons, with no statistically significant peaks or declines.

In contrast to nutritional parameters, carbon footprint values exhibited a small but statistically significant seasonal difference (*p* = 0.032). The mean carbon footprint values were highest in autumn (0.820 ± 0.529 kg CO₂e) and lowest in summer (0.704 ± 0.552 kg CO₂e), with intermediate values in spring (0.719 ± 0.551 kg CO₂e) and winter (0.796 ± 0.537 kg CO₂e). The total water footprint values did not differ significantly across seasons (*p* = 0.654). The mean water footprint values were similar in spring (0.268 ± 0.804 L), summer (0.236 ± 0.715 L), autumn (0.197 ± 0.642 L), and winter (0.208 ± 0.667 L).

### 3.3 Seasonal differences in environmental footprints across meal groups

The analysis revealed significant variation in carbon footprint values across meal categories in all seasons (*p <* 0.001). This finding indicates that greenhouse gas emissions were predominantly influenced by meal type rather than seasonal variation (see Table 4). This pattern remained consistent throughout the year, indicating that the intrinsic characteristics of food groups exert a dominant influence on the shaping of emission profiles. A statistically significant variation in carbon footprint values was observed across different meal categories within each season (*p <* 0.001). Across seasons, pastries, yogurt-based dishes, desserts, and meat-based meals were consistently ranked among the highest-emission categories. During the summer months, the mean carbon footprints for pastries, yogurt-based dishes, desserts, and meat-based meals were recorded as 1.126 ± 0.444 kg CO_2_e, 1.351 ± 0.236 kg CO_2_e, 1.298 ± 0.758 kg CO_2_e, and 1.232 ± 0.322 kg CO_2_e, respectively. High values were also observed in winter, particularly for pastries (1.589 ± 0.496 kg CO₂e), yogurt-based dishes (1.377 ± 0.251 kg CO₂e), and desserts (1.416 ± 0.653 kg CO₂e). Pairwise comparisons showed that these high-emission groups differed significantly from the majority of low-emission, plant-based categories (*p <* 0.05). In contrast, salads, legumes, vegetable dishes prepared with olive oil, and compotes consistently recorded the lowest carbon footprint values across seasons, generally ranging between 0.10 and 0.40 kg CO₂e per portion. Meal groups exhibiting intermediate emissions (e.g., rice and pasta dishes) showed minimal seasonal fluctuation, and these variations were predominantly non-statistically significant. In the context of olive oil-based dishes and compotes, seasonal fluctuations were observed in specific menu cycles, reflecting their limited representation, without any discernible alteration in the prevailing ranking patterns.

**Table 4:** Seasonal variations of carbon footprint values by meal groups.

Meal Group	Summer		Autumn		Winter		Spring	
	Meal (n)	Carbon footprint	Meal (n)	Carbon footprint	Meal (n)	Carbon footprint	Meal (n)	Carbon footprint
Soups	42	0.48 ± 0.43	36	0.56 ± 0.45	45	0.62 ± 0.45	43	0.71 ± 0.50
Meatballs	13	1.16 ± 0.45	10	1.14 ± 0.47	9	1.28 ± 0.40	15	0.94 ± 0.27
Meat and veggies	4	0.64 ± 0.39	7	0.81 ± 0.33	8	0.59 ± 0.28	3	0.80 ± 0.00
Pastas	18	0.76 ± 0.32	20	0.77 ± 0.30	15	0.84 ± 0.14	18	0.76 ± 0.30
Salads	19	0.32 ± 0.39	27	0.23 ± 0.15	25	0.22 ± 0.13	32	0.20 ± 0.25
Meat dishes	20	1.23 ± 0.32	18	1.03 ± 0.31	13	1.21 ± 0.29	18	1.07 ± 0.23
White meats	15	0.81 ± 0.48	25	0.99 ± 0.52	24	1.01 ± 0.44	19	0.80 ± 0.48
Legumes	7	0.15 ± 0.14	4	0.17 ± 0.15	6	0.17 ± 0.14	6	0.13 ± 0.13
Garnishes	21	0.36 ± 0.17	20	0.34 ± 0.19	20	0.33 ± 0.19	15	0.37 ± 0.22
Pastries	5	1.13 ± 0.44	7	1.50 ± 0.53	9	1.59 ± 0.50	8	1.55 ± 0.51
Yogurt	24	1.35 ± 0.24	27	1.33 ± 0.25	25	1.38 ± 0.25	21	1.31 ± 0.22
Desserts	17	1.30 ± 0.76	21	1.37 ± 0.64	17	1.42 ± 0.65	21	1.26 ± 0.76
Pilafs	24	0.28 ± 0.39	21	0.82 ± 0.46	28	0.59 ± 0.42	23	0.31 ± 0.36
Olive oil dishes	3	0.20 ± 0.01	1	N/A	3	0.39 ± 0.34	7	0.28 ± 0.22
Compotes	7	0.21 ± 0.11	2	N/A	4	0.65 ± 0.24	4	0.10 ± 0.10
Fruits	22	0.55 ± 0.25	22	0.78 ± 0.25	25	0.66 ± 0.22	14	0.60 ± 0.29
*p*-value	*P <* 0.001		*P <* 0.001		*P <* 0.001		*P <* 0.001	

One-Way ANOVA; *post-hoc*: Games–Howell, *p <* 0.05.

**Figure 3:**
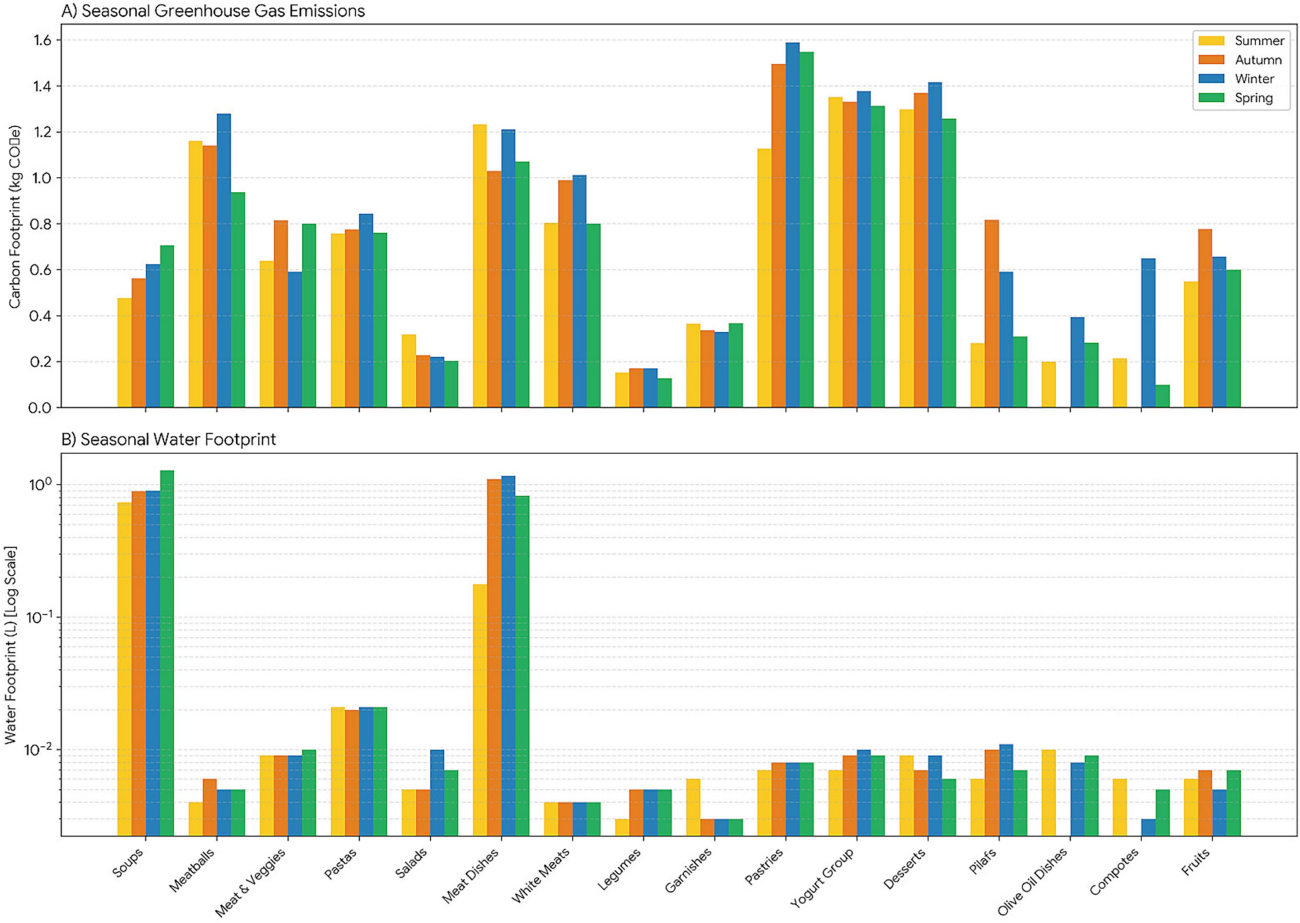
Seasonal variations in environmental footprints across meal groups. Comparative analysis of **(A)** carbon footprint (kg CO₂-eq) and **(B)** water footprint (L) across four seasonal menu cycles. The grouped bar design allows direct comparison of seasonal variability within each meal category and illustrates whether relative environmental ranking patterns remain stable or shift across seasons. Bar heights represent mean environmental impact values for each meal group within each season (Summer, Autumn, Winter, and Spring). **(B)** Utilizes a logarithmic scale to facilitate interpretation across a wide range of water footprint values.

When the carbon footprint was evaluated at the aggregate level, a modest seasonal effect was detected (*p* = 0.032; Table 2). The mean values were found to be similar in spring (0.719 ± 0.551 kg CO₂e), summer (0.704 ± 0.552 kg CO₂e), and autumn (0.820 ± 0.529 kg CO₂e), and remained elevated in winter (0.796 ± 0.537 kg CO₂e). A direct comparison between the two seasons did not reveal any significant differences. Stratified analyses across 1,048 dishes in 16 meal categories and four seasonal menu cycles, corroborated pronounced group-level differences within each season (see Table 4). For instance, in spring, desserts (1.257 kg CO₂e) and yogurt-based dishes (1.313 kg CO₂e) exhibited the highest average emissions, while olive oil–based vegetable dishes (0.282 kg CO₂e) and compotes (0.100 kg CO₂e) demonstrated the lowest. This relative ordering remained largely stable across seasons, with only minor shifts (Figure 3A).

Water footprint values differed significantly across meal groups within each season (*p <* 0.001; Table 5). Across all seasons, soups consistently exhibited the highest water footprint values. The mean water usage for soups ranged from 0.731 ± 1.280 L in summer to 1.280 ± 1.554 L in spring, with similarly elevated values observed in autumn (0.895 ± 1.344 L) and winter (0.902 ± 1.254 L). The investigation revealed that meat-based dishes generally had the second-highest water footprint values. However, the magnitude of these values varied across seasons. Lower values were observed in summer (0.177 ± 0.866 L), whereas substantially higher averages were recorded in autumn (1.099 ± 0.719 L) and winter (1.166 ± 0.945 L), with spring values remaining elevated (0.829 ± 0.633 L). A significant divergence was observed between these meal groups and the majority of other categories (see Table 5).

**Table 5:** Seasonal variations of water footprint values by meal groups.

Meal Group	Summer		Autumn		Winter		Spring	
	Meal (*n*)	Water footprint	Meal (*n*)	Water footprint	Meal (*n*)	Water footprint	Meal (*n*)	Water footprint
Soups	42	0.731 ± 1.280	36	0.895 ± 1.344	45	0.902 ± 1.254	43	1.280 ± 1.554
Meatballs	13	0.004 ± 0.003	10	0.006 ± 0.004	9	0.005 ± 0.004	15	0.005 ± 0.005
Meat and veggies	4	0.009 ± 0.003	7	0.009 ± 0.005	8	0.009 ± 0.002	3	0.010 ± 0.000
Pastas	18	0.021 ± 0.006	20	0.020 ± 0.005	15	0.021 ± 0.006	18	0.021 ± 0.005
Salads	19	0.005 ± 0.005	27	0.005 ± 0.003	25	0.010 ± 0.011	32	0.007 ± 0.005
Meat dishes	20	0.177 ± 0.866	18	1.099 ± 0.719	13	1.166 ± 0.945	18	0.829 ± 0.633
White meats	15	0.004 ± 0.002	25	0.004 ± 0.002	24	0.004 ± 0.001	19	0.004 ± 0.002
Legumes	7	0.003 ± 0.002	4	0.005 ± 0.001	6	0.005 ± 0.000	6	0.005 ± 0.000
Garnishes	21	0.006 ± 0.002	20	0.003 ± 0.002	20	0.003 ± 0.002	15	0.003 ± 0.002
Pastries	5	0.007 ± 0.0004	7	0.008 ± 0.001	9	0.008 ± 0.0005	8	0.008 ± 0.005
Yogurt	24	0.007 ± 0.001	27	0.009 ± 0.001	25	0.010 ± 0.001	21	0.009 ± 0.001
Desserts	17	0.009 ± 0.003	21	0.007 ± 0.003	17	0.009 ± 0.003	21	0.006 ± 0.003
Pilafs	24	0.006 ± 0.004	21	0.010 ± 0.004	28	0.011 ± 0.004	23	0.007 ± 0.001
Olive oil dishes	3	0.010 ± 0.003	1	N/A	3	0.008 ± 0.003	7	0.009 ± 0.003
Compotes	7	0.006 ± 0.003	2	N/A	4	0.003 ± 0.002	4	0.005 ± 0.000
Fruits	22	0.006 ± 0.005	22	0.007 ± 0.006	25	0.005 ± 0.003	14	0.007 ± 0.002
*p*-value	*P <* 0.001		*P <* 0.001		*P <* 0.001		*P <* 0.001	

One-Way ANOVA; *post-hoc*: Games–Howell, *p <* 0.05.

In contrast, the majority of meal groups – including white meat dishes, pasta, legumes, salads, side dishes, pastries, yogurt-based dishes, desserts, pilafs, olive oil-based dishes, compotes, and fruits – clustered at substantially lower water footprint levels across all seasons. For the groups in question, water utilization generally remained within a narrow range of approximately 0.003–0.021 L, with minimal seasonal variation. A comparison of these lower-impact groups revealed minimal differentiation. For specific meal categories, including olive oil-based dishes and compotes, missing values were observed in certain seasons because these dishes were infrequently included in the menu cycle. These gaps were minimal and did not significantly affect the overall distribution or interpretation of water footprint values (Figure 3B).

### 3.4 Relationships between nutritional content and environmental impact

Spearman correlation analysis demonstrated statistically significant positive associations between energy, protein, and fat content and both carbon and water footprints (Table 6).

**Table 6:** The relationships between the carbon and water footprints of foods and their energy and specific nutrient values.

Variables	Energy (kcal)	Carbohydrate (g)	Protein (g)	Fat (g)	Carbon footprint (kg CO₂e)	Water footprint (L)
Energy (kcal)	–	0.627***	0.733***	0.864***	0.202***	0.229***
Carbohydrate (g)		–	0.224***	0.308***	0.012 (ns)	0.147***
Protein (g)			–	0.716***	0.218***	0.196***
Fat (g)				–	0.252***	0.322***
Carbon footprint (kg CO₂e)					—	0.391***
Water footprint (L)						—

***p < 0.001; ns: not significant. Spearman’s rho coefficients reported.

The correlation between energy intake and both the carbon footprint (*ρ* = 0.202) and the water footprint (*ρ* = 0.229) was weak to moderate. The protein content exhibited a positive correlation with both the carbon footprint (*ρ* = 0.218) and the water footprint (*ρ* = 0.196). The correlation between fat content and the water footprint (*ρ* = 0.322) was more substantial than that with the carbon footprint (*ρ* = 0.252). In contrast, the carbohydrate content exhibited no significant correlation with the carbon footprint (*ρ* = 0.012, *p* = 0.704) and demonstrated only a weak association with the water footprint. Furthermore, a moderate correlation was observed between carbon and water footprints (*ρ* = 0.391, *p <* 0.001).

### 3.5 Nutritional equivalence-based comparison of carbon and water footprints across meal groups

After the initial per-portion LCA calculations, carbon and water footprints were further examined under nutritionally equivalent conditions by adjusting for energy and protein content and by expressing impacts per unit of energy and per unit of protein (see Table 7). This was achieved by adjusting for energy and protein content, and by expressing environmental impacts per unit of energy and protein (see Table 7). For the ANCOVA-based adjusted estimates, some water footprint values were negative due to statistical adjustment for covariates; these values do not indicate negative physical impacts, but reflect relative positioning of meal groups after energy and protein adjustment. Following adjustment for both energy and protein intake, a significant variation in carbon footprint was observed across the various meal groups (*p <* 0.001). The study found that the carbon footprint of salad, legume, compote, and olive oil-based dishes was the lowest, while that of yogurt-based dishes, pastries, meatballs, desserts, and white meat dishes was the highest.

**Table 7:** Nutritional equivalence-adjusted and normalized carbon and water footprints across meal groups.

Meal group	Carbon footprint			Water footprint		
	ANCOVA-adjusted carbon footprint estimate (kg CO₂e)†	Carbon footprint per g protein (kg CO₂e/g protein)‡	Carbon footprint per Kcal (kg CO₂e/kcal) §	ANCOVA-adjusted water footprint estimate (L)	Water footprint per g protein (L/g protein) ‡	Water footprint per Kcal (L/kcal) §
Soups	0.576	0.146 ^a^	0.004 ^a^	0.897	0.247^A^	0.006 ^E^
Meatballs	1.201	0.053 ^ab^	0.003 ^ab^	0.375	0.0002^B^	0.0001^B^
Meat and veggies	0.805	0.055 ^ab^	0.004 ^a^	0.200	0.0007^BC^	0.0005^C^
Pastas	0.709	0.157 ^a^	0.003 ^ab^	−0.107	0.004^D^	0.0009^D^
Salads	0.235	0.250 ^cd^	0.004 ^a^	−0.141	0.005^D^	0.001^D^
Meat dishes	1.147	0.060 ^ab^	0.003 ^ab^	1.457	0.068^E^	0.003^E^
White meats	1.168	0.040 ^be^	0.004 ^a^	0.623	0.0002^B^	0.0004^C^
Legumes	0.229	0.014 ^e^	0.001^e^	0.218	0.0004^B^	0.0002^B^
Garnishes	0.349	0.163 ^ac^	0.024 ^d^	−0.117	0.003^D^	0.0008^D^
Pastries	1.369	0.409 ^f^	0.009 ^c^	−0.159	0.002^C^	0.0004^C^
Yogurt	1.404	0.265 ^cd^	0.057 ^f^	−0.010	0.002^C^	0.0004^C^
Desserts	1.177	0.333 ^d^	0.004 ^a^	−0.204	0.002^C^	0.0003^C^
Pilafs	0.383	0.076 ^ab^	0.002 ^eb^	−0.128	0.001^C^	0.0003^C^
Olive oil dishes	0.285	0.065 ^ab^	0.002 ^eb^	−0.057	0.002^C^	0.0006^C^
Compotes	0.242	0.312 ^d^	0.002 ^eb^	−0.232	0.026^E^	0.0007^C^
Fruits	0.647	0.772 ^g^	0.009 ^c^	−0.167	0.007^F^	0.001^D^

^†^ANCOVA adjusted for energy and protein. ^‡^One-way ANOVA with Games–Howell post hoc. ^§^One-way ANOVA with Games–Howell post hoc. Different superscript letters within the same column indicate statistically significant differences between meal groups based on post-hoc comparisons. Negative adjusted values may occur because ANCOVA-derived estimates represent model-based covariate-adjusted means rather than directly observed physical footprint values (113–117). These estimates are statistical constructs used for comparative interpretation within the model framework and should not be interpreted as literal negative environmental impacts (116, 117). In particular, adjusted means may fall below zero as a result of the covariate adjustment process, especially when distributions are highly skewed or when the relationship between covariates and outcomes is strong (113, 114). Accordingly, such values reflect the statistical adjustment procedure rather than actual negative physical or environmental outcomes (113, 116, 117).

When the carbon footprint was normalized per gram of protein, significant heterogeneity among the meal groups was observed (*p <* 0.001). Legume-based dishes exhibited the lowest carbon footprint per unit of protein, with white meat and mixed meat dishes ranking second and third, respectively. Conversely, the carbon footprint per gram of protein in yogurt-based products, desserts, pastries, and fruits was found to be substantially higher. A marked difference was observed between the fruit and all other meal groups, indicating the former’s low protein content. Energy-normalized carbon footprint analysis yielded consistent results (*p <* 0.001). Despite comparable caloric content, marked differences in carbon footprint were observed among the meal groups. The analysis revealed that dishes containing legumes had the lowest energy-adjusted carbon footprints, while meals containing dairy products and composite dishes had higher carbon footprints. A similar pattern was observed for water footprint outcomes. Following adjustment for energy and protein intake, a significant difference in water footprint was observed across meal groups (*p <* 0.001). In nutritionally equivalent conditions, meat-based dishes, soups, and white meat dishes exhibited the highest water footprint values, while salads, legumes, garnishes, pasta-based dishes, olive oil–based meals, and compotes clustered at lower levels.

**Figure 4:**
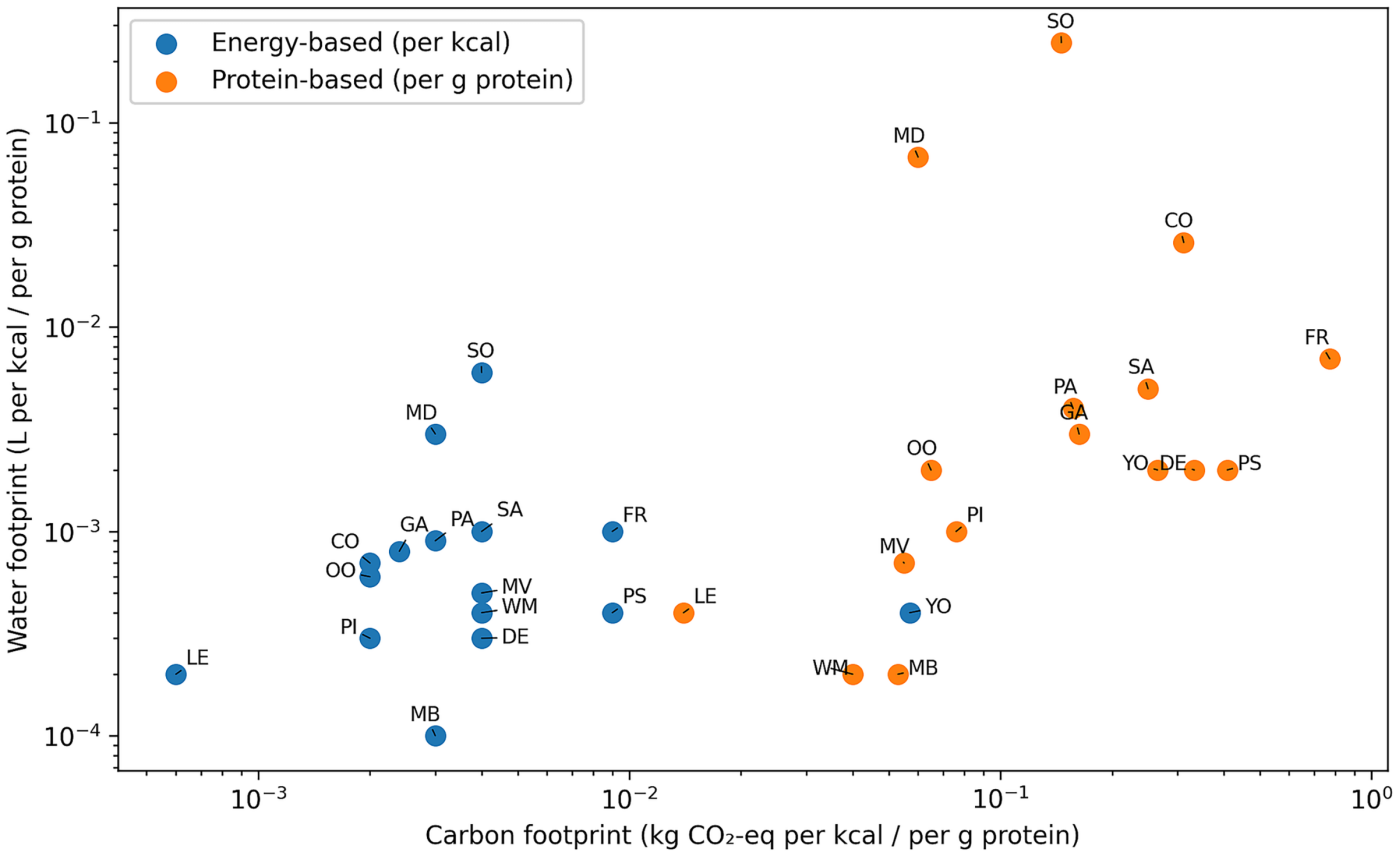
Comparative distribution of carbon and water footprints across meal groups after normalization by energy and protein content. Carbon footprint (kg CO₂-eq per kcal and per g protein) and water footprint (L per kcal and per g protein) are presented for each meal group under two functional units: energy-based (per kcal, blue) and protein-based (per g protein, red). Both axes are displayed on a logarithmic scale to enable comparison across a wide range of environmental impact values. To improve interpretability, labels are slightly offset and connected to their corresponding points. The figure illustrates how meal groups shift in relative position depending on whether environmental performance is evaluated on an energy-normalized or protein-normalized basis. SA, salads; LE, legumes; OO, olive oil dishes; CO, compotes; PI, pilafs; GA, garnishes; SO, soups; FR, fruits; PA, pastas; MV, meat and vegetable dishes; MD, meat dishes; WM, white meats; MB, meatballs; DE, desserts; PS, pastries; YO, yogurt group.

Protein-normalized water footprint analysis revealed significant differences among the meal groups (*p <* 0.001). Legumes and white meat dishes demonstrated the most economical use of water per gram of protein, while meat-based dishes exhibited higher values. Furthermore, a high correlation was observed between the water footprint of soups and their protein content. Energy-normalized water footprint analysis further demonstrated persistent differences among meal groups (*p <* 0.001). A comparison of the two normalization approaches demonstrated that meal groups occupied various positions depending on whether impacts were expressed per unit of energy or per unit of protein. The application of energy-based normalization led to the aggregation of meal groups, whereas protein-based normalization accentuated the disparities between groups, particularly for meals with minimal protein content (Figure 4).

In the energy-normalized (per kcal) comparison, most meal groups clustered in the lower-left region of the plot. This observation reflects relatively low, closely grouped carbon and water footprint values when evaluated on an equal energy basis. Under the prevailing conditions of this study, a reduction in environmental differences was observed between the various meal groups. Legumes (BA), pilafs (PI), and several plant-based meal categories exhibited low energy-adjusted carbon and water footprints. Conversely, soups (SO) and specific mixed-content meals exhibited distinct separation from this cluster, predominantly attributable to elevated water footprint values despite energy normalization (Figure 4).

The evaluation of meals on a protein-normalized basis (per g protein) shifted the distribution toward the upper-right region of the plot. Consequently, the differences between the meal groups became more evident. It was observed that meal categories exhibiting low protein density, such as fruits (FR), compotes (CO), desserts (DE), and yogurt-based dishes (YO), demonstrated higher carbon and water footprints per unit of protein. In contrast, the consumption of legumes (BA) and meatballs (MB) remained within a lower-impact range following protein normalization.

## 4 Discussion

### 4.1 Meal composition as the primary driver of environmental impact

A fundamental step toward developing a more sustainable menu planning strategy is identifying the factors that shape the environmental performance of institutional food services. The findings of this study suggest that the carbon and water footprints of meals served in university canteens are predominantly determined by the composition of food groups within those meals. Despite seasonal variability, which introduces statistically detectable differences, these fluctuations are secondary in comparison to the predominant influence of meal composition on overall environmental burden.

This finding aligns with extant evidence that the nature of the food produced and consumed is the predominant factor determining food-related greenhouse gas emissions. André et al. (71) explicitly identify meal composition as the primary driver of emissions originating from food systems, a finding that closely aligns with the outcomes observed in the present study.

Within our analysis, meals containing animal-based components—particularly red meat–based main dishes and dairy-containing groups—exhibited higher carbon and water footprint values across both absolute and normalized assessments. Comparable conclusions were reported by Campobasso et al. (39), who demonstrated that environmental impacts in a university canteen in southern Italy were primarily shaped by food content and weekly serving frequency. In that study, menus centered on meat and salmon were identified as having the highest environmental impact, with the cumulative effect amplified when high-impact animal-based foods were served more frequently. The elevated footprint of salmon-based menus was attributed to transportation requirements, whereas red meat–based menus showed high environmental loads from the earliest stages of the production chain (39).

These observations are consistent with previous assessments showing that animal-based production is associated with higher greenhouse gas emissions and greater resource use than plant-based alternatives (59, 72, 73). Global evidence similarly indicates that livestock production constitutes a major share of food-system emissions, with ruminant-based foods standing out as particularly high-impact contributors (30–32, 59, 74–76). Taken together, these findings support the interpretation that the higher impacts observed in the present study are driven primarily by the presence and frequency of animal-based meal components rather than by seasonal menu variation alone. A similar pattern is evident for water footprint outcomes. Animal-based foods, especially meat-based meals, are consistently reported to require substantially more freshwater than plant-based alternatives because of feed irrigation and livestock-related water demand (15, 33, 77, 78). Institutional food-service studies likewise show that increases in meat content are associated with higher water footprint values, whereas plant-based substitutions can reduce environmental burdens substantially (79–82). In contrast, the present study found that legume-based meals, rice dishes, and olive oil-based vegetable preparations were associated with the lowest carbon and water footprint values. This observation is consistent with earlier research that emphasized the potential of plant-based protein sources to provide both environmental and nutritional benefits (3, 19, 81, 83, 84). In particular, the low protein-adjusted carbon and water footprints of legumes highlight their potential role as a cornerstone of sustainable menu planning.

The findings, when considered collectively, indicate that efforts to mitigate environmental impacts within institutional food services are likely to be more effective when centered on restructuring meal composition rather than relying solely on seasonal adjustments (45, 71, 85). Evidence from Türkiye supports this conclusion, as demonstrated in studies by Oruçoğlu et al. (4) and Yeşildemir (45), which show that increases in the animal protein and saturated fat content of university menus are accompanied by marked rises in both carbon and water footprints.

### 4.2 Seasonal variability: limited influence compared to meal type

Carbon and water footprints in institutional food services can also vary with seasons. Yet, it is not seasonality per se that causes the variability, but rather differences at the menu level or in the production/supply gradient. In food and meal choices, seasonal differences in carbon and water footprints are influenced by how the transitions in consumed food groups, off-season production modes, and energy intensities interact. Therefore, the impact of seasonality on environmental impacts is indirectly related (34, 86, 87). Moreover, when seasonal footprints are considered, both Greenhouse Gas Emissions GHGe and water footprints of institutional menus can be higher in spring and lower in winter (GHGe: 227.5 vs.178.9 kg CO_2_e; water: 167,663 vs.146,733 m^3^/ton), with most of these differences due to the change in seasonality and offered meat-based dishes (87). However, while seasonal studies show that carbon footprints may differ in a statistically significant way, it is generally thought that the extent of this variation is quite constrained.

Season also primarily affects environmental footprints through changes in menu and purchasing patterns, and is not a stand-alone factor. In Italy, a study in a long-term care facility found that the GHGe of spring/summer compared to autumn/winter menus was slightly lower (2.64 vs. 2.82 kgCO₂e/meal), due to differences in animal products and portion size (88). On the contrary, in Italian school canteens, winter menus were characterized by a higher carbon footprint than summer menus because a greater amount of meat and dairy products was consumed during cold months, whereas there was less consumption of these foods in summertime; summer menus included more fresh vegetables, fruit, and cereals, whose production has a lower carbon impact (34). Between studies, beef and red meat often stand out as the main drivers of both carbon and water footprints (86–90). Consistent with these results, previous analyses of institutional catering have also shown that seasonality plays a limited role in determining environmental impacts, while the selection of food groups was the main influencing factor (34, 73).

Out-of-season production methods can also increase the environmental burden, especially for fruit and vegetables. Produce grown in heated greenhouses during the off-season will generally have carbon footprints 14–46% higher than those of open-field, seasonal produce (86, 91). The high energy use and industrial inputs required to maintain greenhouse temperatures contribute to emissions and water use (91). Such additional system-wide effects were also observed in sensitivity analyses using the Environmental Analysis Tool for School Meals (EATS), which showed that off-season production of fruits and vegetables in heated greenhouses significantly increases carbon footprints (86). Also, in an Italian menu inspired by two other Mediterranean diets, replacing fresh fruit and vegetables with frozen or imported items, as well as seasonal fruits and greenhouse-grown crops, resulted in higher carbon footprints (92).

Food’s water footprint is also highly dependent on where and when it was grown. For example, the import of certain vegetables during summer months in Spain may result in saving water, while imports at other times usually entail increased carbon and water footprints (93). Furthermore, regardless of seasonal considerations, animal-derived foods always have an order-of-magnitude higher water footprint than plant-based options (4). Monthly analyses of fruit and vegetable consumption in Spain showed that local, seasonal production decreased the footprint for some products, but in other months imports were associated with lower footprints (93, 94). According to one Spanish study, the lowest observed carbon footprint (August) could be explained by decreased appetite or lower food intake during warmer periods; in contrast, household-level carbon and water footprints were higher at the end of the year due to a higher consumption of meat, fish/seafood, and desserts (95).

In the current investigation, although significant differences in carbon footprints were found, the overall range of variation was low. The slight variations in energy, protein, and macronutrient composition across the year might be due to standardized menu planning strategies in university canteens, which help compensate for seasonal variations. A marginally greater CO_2_e per subdivision was seen in the autumn and winter, which may be associated with increased provision of animal-based and fried dishes on these days, but there were insufficient changes to affect the overall trend. Generally, the results reinforce menu composition over seasonality as a more determinative factor in environmental sustainability.

In conclusion, the impact of menus depends not only on food choices but also on the season and the mode of production (natural cycle vs. greenhouse). However, for institutional menus, the limited evidence so far suggests that seasons primarily impact environmental footprints by influencing menu composition (such as the red meat/vegetable ratio) and procurement practices (seasonal versus greenhouse products, local versus imported products) rather than acting as a direct driver. As such, seasonality by itself does not ensure reduced environmental impacts; instead, on average, menus that are both predominantly plant-based and seasonal and have regional relevance tend to have lower footprints (86, 87). This emphasizes that sustainability-focused interventions cannot rely on a ‘seasonal menu’ approach but should instead adopt content-oriented strategies with a higher likelihood of impact.

### 4.3 Nutritional–environmental trade-offs and the role of normalization

A key contribution of this study lies not in redefining the functional unit of the LCA, but in complementing the primary portion-based assessment with energy- and protein-based normalization. This distinction is important because the LCA results were generated per standardized serving, whereas nutritional metrics were used analytically to enable function-oriented comparisons across meal groups. A significant contribution of this research lies in its approach to normalization, specifically evaluating carbon and water footprints relative to energy and protein content. While most existing literature assesses environmental impacts on a per-portion or per-meal basis, these studies often overlook the critical factor of nutritional equivalence (34, 39, 43, 45, 90, 91).

According to the literature, calculating carbon and water footprints per unit of protein or per unit of energy is considered a more accurate and realistic approach, as it places the primary function of food—its nutritional provision—at the center of environmental assessment (96–98).

In the current study, energy-normalized results showed that the environmental footprints of various meal groups converged (Table 7), suggesting that, when meals of equivalent energy were compared, differences in ecological effects tended to decrease. However, it has also been reported that assessments based exclusively on mass (kg) can be misleading because, under this approach, foods with low energy density (e.g., vegetables) could appear environmentally preferable. Yet, their carbon footprint per kilocalorie may rival that of animal products (99–101).

The inclusion of protein-based normalization further highlighted the environmental pressure exerted by animal products. The idea of protein delivery efficiency can help explain this observation. This is the protein produced per unit of environmental burden and is a key indicator in sustainability assessments (97). In plant-based protein sources (legumes and cereals), increased protein content is correlated with greater production. In contrast, proteins derived from animal sources, in particular beef, were observed to have relatively higher costs associated with carbon and water used per unit of protein (90, 97, 102).

In the present work, legume-containing meals were found to have lower-protein-adjusted carbon and water footprints, indicating that these foods are healthier and more sustainable than other protein sources. Our findings agree with the existing literature, which underlines the relationship between protein quality and environmental impact (5, 81, 92, 97, 100, 102). The existing literature also suggests that legumes in general are roughly 250 times more efficient than beef at generating greenhouse gases per gram of protein, and that soybeans specifically supply protein and energy equivalent to most meat products, with an overall lower cradle-to-plate environmental load (81, 102, 103). On the other hand, food groups with low protein content and high energy density (e.g., desserts, compotes, and some dairy products) had higher environmental footprints per unit of protein. This observation implies that assessments based solely on portion- or energy-based metrics may not fully capture the nutrition-environment nexus.

The results show that selecting the functional unit (e.g., 100 g, 100 kcal, or protein-based) can significantly alter outcomes, and policy implications can differ accordingly (100, 101). To the best of our knowledge, the most complete approach would be to use nutrient density indices, such as NRF 9.3, which take into account not only energy and protein, but also vitamins, minerals, and dietary fiber, in this context. These indices enable a more comprehensive assessment by incorporating general nutritional quality and environmental burden into a single score (98). Therefore, in sustainable menu planning, nutrition-equivalence-based assessment methods are a more realistic and practicable framework for policymakers and institutional food service managers. However, although animal-derived foods such as beef and lamb have been claimed to have a high carbon footprint and water use, the literature suggests that protein and energy requirements can be met at dramatically lower environmental costs through the efficient application of plant-derived substitutions and food swaps (104, 105). Some of these strategies include the use of legume–whole grain combinations to increase protein quality (81, 97), the substitution of red meat with poultry or sustainably harvested fish to achieve a reduction in carbon footprints by approximately 35% (57, 73, 82), and removal of processed meats for rapid health and environmental benefits (43) as well as adoption of dietary patterns such as Mediterranean and Atlantic diets which reposition animal-based foods on the periphery rather than core elements (64, 81, 92). The results of this study show that considering the environmental impact of food, in terms of both quantity and nutritional quality, provides more robust, scientifically grounded evidence on which sustainable menu planning can be based.

### 4.4 Implications for sustainable menu planning in university canteens

From a practical institutional food-service perspective, the present findings suggest that sustainability-oriented menu revision should prioritize meal composition as the main intervention point. In operational terms, this means that catering managers can use environmental assessment not only to identify high-impact meals, but also to redesign menu cycles by reducing the frequency of high-impact components and increasing the availability of lower-impact nutritionally relevant alternatives. In this respect, the results provide a decision-support basis for routine menu planning in university canteens.

The results show that sustainability-driven menu planning in university canteens is an effective intervention for reducing environmental burdens. More specifically, there is growing evidence to highlight that increased availability of legumes, cereals, and olive oil-based vegetable dishes in meal options may be associated with lower environmental footprints while meeting nutritional requirements.

According to this model, the most effective way to reduce the environmental impact of university menus seems to be changing the food groups consumed rather than how they are produced (106). Unlike the total elimination of animal-derived products, this study showed that judicious, rational use can offset environmental impacts. Foods of ruminant origin, such as beef and lamb, have been reported to have the highest GHGe per unit of protein and water usage. As a result, reductions in portion size or the number of intakes were estimated to account for 14 to 46% of the decrease in GHGe (75, 106). Reductions in meat frequency and serving size may be an effective means of achieving substantial environmental benefits in institutional food services, as suggested by prior research (59, 71–73, 105).

In this respect, increased inclusion of legumes, cereals, and olive oil-based vegetable dishes is fundamental not only for environmental sustainability but also to safeguarding a nutritionally adequate diet. Legumes, nuts, and whole grains have low carbon and water footprints and form the basis for food-swap strategies to meet protein needs in university menus (104, 105). In addition, transitioning away from red meat to poultry or sustainably sourced seafood could be an intermediate solution that mitigates adverse environmental effects and is acceptable to students (57, 73, 82).

Other positive aspects of menu planning go beyond simply changing the types of food. Baking and boiling were identified in this study as having lower carbon footprints. This also implies that reducing the environmental impact of energy-intensive cooking methods, such as frying, might yield a greater decrease. Simultaneously, the effectiveness of sustainable menus does not rely solely on kitchen actions but also on student choices and behavior. It is suggested that introducing a vegetarian day (“Meat-Free Day”) could lead to a 16.8% decrease in on-campus sales if implemented once per week. In addition, if it were assumed that 8.7% of the student population shifted to off-campus dining on such days, all associated emission offsets could be lost (107). These results underline the relevance of nudging instruments from a perspective of choice architecture, rather than hard limitations. The strategic positioning of low-impact options at the top of the menu, combined with relegating high-carbon items to lower positions, has been shown to elicit balanced and acceptable demand responses (5). Though traffic-light-style climate labels might get consumers’ attention, they alone are not likely to dramatically change behavior in all-you-can-eat systems (108).

Nutritional adequacy is also necessary, along with environmental sustainability. The present study has shown that menus should be evaluated on a per-energy or per-protein-offering basis, rather than on a per-gram basis (98). For some university menus, an imbalance in the recommended energy range (640–880 kcal) has been observed, potentially leaving some micronutrients, such as calcium, incomplete (45). Second, vegetarian diets that overcompensate for the lack of protein with refined grains may be more environmentally friendly than omnivorous diets but may also have adverse health effects (107).

Importantly, the implications of normalized results differ from those derived from per-meal footprint estimates alone. Per-portion environmental values are directly relevant for identifying the immediate environmental burden of meals as served in institutional settings. By contrast, energy- and protein-normalized indicators provide a function-oriented perspective by showing how efficiently different meal groups deliver nutritional value relative to environmental impact. As a result, institutional decision-makers may reach different conclusions depending on whether the primary objective is to reduce the total environmental load of menu cycles or to improve the nutritional efficiency of lower-impact meal alternatives. This distinction is particularly relevant for university catering systems, where operational feasibility and nutritional adequacy must be considered together.

Finally, eating should encompass both a seasonal and a locavore approach to menu planning as part of lifestyle practice. An increase in carbon footprint (up to 46%) for vegetables grown in heated greenhouses, compared with open-field production, confirms that promoting local and seasonal products is crucial (86, 101, 104). Equally, the fact that the volume of food waste in university cafeterias is 7–14% suggests the need to increase interest in vegetable-based side dishes and to include food waste treatment as part of sustainable menu planning (43, 45).

Overall, the findings suggest that the most effective university canteen interventions are those that combine a gradual reduction in high-impact red meat dishes with a greater routine inclusion of plant-based alternatives, while also considering seasonality, procurement practices, and waste management (90). Such an approach is more likely to support sustainability targets when environmental burden, nutritional adequacy, and institutional feasibility are addressed together.

### 4.5 Strengths and limitations

One of the strengths of this paper is that it analyzes the entire menu set from an actual university cafeteria system and uses a thorough life-cycle assessment. Furthermore, the normalization for adjustment energy and protein has addressed a major methodological shortcoming present only in some studies in the literature. However, the study has limitations. A part of the LCA data used is imported from databases in other countries because insufficient data are available for Türkiye. This may introduce context-related uncertainty into the absolute carbon and water footprint estimates, as some background processes may not fully reflect local production conditions, supply chains, or resource-use profiles (109, 110). However, because the same modeling framework and data selection logic were applied consistently across all recipes, the relative comparisons between meal groups are expected to remain more robust than the absolute values (111, 112).

In addition, the harmonized treatment of cooking energy inputs, used to ensure comparability across recipes prepared with different kitchen technologies, may have introduced further uncertainty into the absolute contribution of the cooking stage. In addition, post-consumer waste was not included in the system boundary; therefore, the findings reflect the environmental burdens of meal provision up to the kitchen stage rather than the full impacts associated with consumption and disposal. Furthermore, no formal uncertainty or sensitivity analysis was conducted. As a result, the robustness of the absolute footprint estimates under alternative modeling assumptions, data selections, or proxy substitutions could not be quantitatively tested within the scope of the present study.

## 5 Conclusion

The analysis indicates that university food provision has a significant ecological footprint, and seasonal menu variations affect sustainability. For instance, local sourcing and reduced transportation emissions during certain seasons can significantly lower the environmental impact. The study highlights that red meat, and dairy foods have substantial carbon and water impacts. In contrast, plant-based substitutes, including legumes, meet nutritional targets while being less environmentally damaging. Including protein and energy normalization values clarified the effects of seasonal variation and of production systems, such as greenhouse culture, on sustainability by providing measurable data points.

Nevertheless, it is essential to note that the influences of seasonal variations and production systems become apparent only after the initial selection of basic ingredients. As such, efforts to promote sustainability through institutional meal service should focus on dietary composition. Such interventions could involve reducing the frequency of consumption of ruminant animal flesh and employing strategies such as ‘choice architecture,’ which subtly guides consumer choices toward less resource-intensive foods. Adopting sustainable menus in higher education dining, supported by studies showing a 30% reduction in GHGe emissions from plant-based diets, demonstrates that environmentally friendly and nutritionally sound nourishment is achievable.

## Data Availability

The raw data supporting the conclusions of this article will be made available by the authors, without undue reservation.
